# Supplementary material for: Overlapping cell population expression profiling and regulatory inference in C. elegans
Source: BMC Genomics. 2016 Feb 29;17:159. doi: 10.1186/s12864-016-2482-z (PMC4772325; doi:10.1186/s12864-016-2482-z)
Supplement: Additional file 13: — Web supplement. (DOC 21 kb) [file 12864_2016_2482_MOESM13_ESM.zip › sortWeb/clusters/hier.300.clusters/39.html]

Cluster 39 

## Cluster 39

### Expression

| cnd-1 rep. 1 | cnd-1 rep. 2 | cnd-1 rep. 3 | pha-4 rep. 1 | pha-4 rep. 2 | pha-4 rep. 3 | ceh-27 | ceh-36 | ceh-6 | F21D5.9 | mir-57 | mls-2 | pal-1 | pros-1 | ttx-3 | unc-130 | hlh-16 | irx-1 | ceh-6 (+) hlh-16 (+) | ceh-6 (+) hlh-16 (-) | ceh-6 (-) hlh-16 (+) | cnd-1 singlets | pha-4 singlets | 0 | 60 | 120 | 150 | 180 | 240 | 330 | 390 | 420 | 480 | 540 | 570 | 600 | 630 | 660 | NAME | Functional description |
| --- | --- | --- | --- | --- | --- | --- | --- | --- | --- | --- | --- | --- | --- | --- | --- | --- | --- | --- | --- | --- | --- | --- | --- | --- | --- | --- | --- | --- | --- | --- | --- | --- | --- | --- | --- | --- | --- | --- | --- |
|  |  |  |  |  |  |  |  |  |  |  |  |  |  |  |  |  |  |  |  |  |  |  |  |  |  |  |  |  |  |  |  |  |  |  |  |  |  | C32F10.4 |  |
|  |  |  |  |  |  |  |  |  |  |  |  |  |  |  |  |  |  |  |  |  |  |  |  |  |  |  |  |  |  |  |  |  |  |  |  |  |  | R09H10.2 |  |
|  |  |  |  |  |  |  |  |  |  |  |  |  |  |  |  |  |  |  |  |  |  |  |  |  |  |  |  |  |  |  |  |  |  |  |  |  |  | *nac-2* | NADC (Na+-coupled dicarboxylate transporter) family |
|  |  |  |  |  |  |  |  |  |  |  |  |  |  |  |  |  |  |  |  |  |  |  |  |  |  |  |  |  |  |  |  |  |  |  |  |  |  | K04A8.1 |  |
|  |  |  |  |  |  |  |  |  |  |  |  |  |  |  |  |  |  |  |  |  |  |  |  |  |  |  |  |  |  |  |  |  |  |  |  |  |  | *cpd-1* | CarboxyPeptidase D family |
|  |  |  |  |  |  |  |  |  |  |  |  |  |  |  |  |  |  |  |  |  |  |  |  |  |  |  |  |  |  |  |  |  |  |  |  |  |  | *tbc-3* | TBC (Tre-2/Bub2/Cdc16) domain family |
|  |  |  |  |  |  |  |  |  |  |  |  |  |  |  |  |  |  |  |  |  |  |  |  |  |  |  |  |  |  |  |  |  |  |  |  |  |  | T16G1.9 |  |
|  |  |  |  |  |  |  |  |  |  |  |  |  |  |  |  |  |  |  |  |  |  |  |  |  |  |  |  |  |  |  |  |  |  |  |  |  |  | *lgc-27* | Ligand-Gated ion Channel |
|  |  |  |  |  |  |  |  |  |  |  |  |  |  |  |  |  |  |  |  |  |  |  |  |  |  |  |  |  |  |  |  |  |  |  |  |  |  | T13H5.6 |  |
|  |  |  |  |  |  |  |  |  |  |  |  |  |  |  |  |  |  |  |  |  |  |  |  |  |  |  |  |  |  |  |  |  |  |  |  |  |  | *svh-2* | Suppressor of VHp-1 deletion lethality |
|  |  |  |  |  |  |  |  |  |  |  |  |  |  |  |  |  |  |  |  |  |  |  |  |  |  |  |  |  |  |  |  |  |  |  |  |  |  | *gly-5* | GLYcosylation related |
|  |  |  |  |  |  |  |  |  |  |  |  |  |  |  |  |  |  |  |  |  |  |  |  |  |  |  |  |  |  |  |  |  |  |  |  |  |  | *rsd-3* | RNAi Spreading Defective (see also sid) |
|  |  |  |  |  |  |  |  |  |  |  |  |  |  |  |  |  |  |  |  |  |  |  |  |  |  |  |  |  |  |  |  |  |  |  |  |  |  | *rga-1* | Rho GTPase Activating protein |
|  |  |  |  |  |  |  |  |  |  |  |  |  |  |  |  |  |  |  |  |  |  |  |  |  |  |  |  |  |  |  |  |  |  |  |  |  |  | *tag-250* | Temporarily Assigned Gene name |
|  |  |  |  |  |  |  |  |  |  |  |  |  |  |  |  |  |  |  |  |  |  |  |  |  |  |  |  |  |  |  |  |  |  |  |  |  |  | *nhr-173* | Nuclear Hormone Receptor family |
|  |  |  |  |  |  |  |  |  |  |  |  |  |  |  |  |  |  |  |  |  |  |  |  |  |  |  |  |  |  |  |  |  |  |  |  |  |  | ZK899.3 |  |
|  |  |  |  |  |  |  |  |  |  |  |  |  |  |  |  |  |  |  |  |  |  |  |  |  |  |  |  |  |  |  |  |  |  |  |  |  |  | *arrd-7* | ARRestin Domain protein |
|  |  |  |  |  |  |  |  |  |  |  |  |  |  |  |  |  |  |  |  |  |  |  |  |  |  |  |  |  |  |  |  |  |  |  |  |  |  | F56A8.9 |  |
|  |  |  |  |  |  |  |  |  |  |  |  |  |  |  |  |  |  |  |  |  |  |  |  |  |  |  |  |  |  |  |  |  |  |  |  |  |  | F54D1.6 |  |
|  |  |  |  |  |  |  |  |  |  |  |  |  |  |  |  |  |  |  |  |  |  |  |  |  |  |  |  |  |  |  |  |  |  |  |  |  |  | *pqn-89* | Prion-like-(Q/N-rich)-domain-bearing protein |
|  |  |  |  |  |  |  |  |  |  |  |  |  |  |  |  |  |  |  |  |  |  |  |  |  |  |  |  |  |  |  |  |  |  |  |  |  |  | F14B8.5 |  |
|  |  |  |  |  |  |  |  |  |  |  |  |  |  |  |  |  |  |  |  |  |  |  |  |  |  |  |  |  |  |  |  |  |  |  |  |  |  | Y4C6B.7 |  |
|  |  |  |  |  |  |  |  |  |  |  |  |  |  |  |  |  |  |  |  |  |  |  |  |  |  |  |  |  |  |  |  |  |  |  |  |  |  | *clec-52* | C-type LECtin |
|  |  |  |  |  |  |  |  |  |  |  |  |  |  |  |  |  |  |  |  |  |  |  |  |  |  |  |  |  |  |  |  |  |  |  |  |  |  | *tsp-17* | TetraSPanin family |
|  |  |  |  |  |  |  |  |  |  |  |  |  |  |  |  |  |  |  |  |  |  |  |  |  |  |  |  |  |  |  |  |  |  |  |  |  |  | F41C3.8 |  |
|  |  |  |  |  |  |  |  |  |  |  |  |  |  |  |  |  |  |  |  |  |  |  |  |  |  |  |  |  |  |  |  |  |  |  |  |  |  | *dhs-25* | DeHydrogenases, Short chain |
|  |  |  |  |  |  |  |  |  |  |  |  |  |  |  |  |  |  |  |  |  |  |  |  |  |  |  |  |  |  |  |  |  |  |  |  |  |  | Y77E11A.2 |  |
|  |  |  |  |  |  |  |  |  |  |  |  |  |  |  |  |  |  |  |  |  |  |  |  |  |  |  |  |  |  |  |  |  |  |  |  |  |  | *tat-4* | Transbilayer Amphipath Transporters (subfamily IV P-type ATPase) |
|  |  |  |  |  |  |  |  |  |  |  |  |  |  |  |  |  |  |  |  |  |  |  |  |  |  |  |  |  |  |  |  |  |  |  |  |  |  | C07H4.1 |  |
|  |  |  |  |  |  |  |  |  |  |  |  |  |  |  |  |  |  |  |  |  |  |  |  |  |  |  |  |  |  |  |  |  |  |  |  |  |  | *nhr-88* | Nuclear Hormone Receptor family |
|  |  |  |  |  |  |  |  |  |  |  |  |  |  |  |  |  |  |  |  |  |  |  |  |  |  |  |  |  |  |  |  |  |  |  |  |  |  | *let-805* | LEThal |
|  |  |  |  |  |  |  |  |  |  |  |  |  |  |  |  |  |  |  |  |  |  |  |  |  |  |  |  |  |  |  |  |  |  |  |  |  |  | *nhr-97* | Nuclear Hormone Receptor family |
|  |  |  |  |  |  |  |  |  |  |  |  |  |  |  |  |  |  |  |  |  |  |  |  |  |  |  |  |  |  |  |  |  |  |  |  |  |  | *cutl-10* | CUTiclin-Like |
|  |  |  |  |  |  |  |  |  |  |  |  |  |  |  |  |  |  |  |  |  |  |  |  |  |  |  |  |  |  |  |  |  |  |  |  |  |  | Y57A10A.26 |  |
|  |  |  |  |  |  |  |  |  |  |  |  |  |  |  |  |  |  |  |  |  |  |  |  |  |  |  |  |  |  |  |  |  |  |  |  |  |  | *arx-7* | ARp2/3 compleX component |
|  |  |  |  |  |  |  |  |  |  |  |  |  |  |  |  |  |  |  |  |  |  |  |  |  |  |  |  |  |  |  |  |  |  |  |  |  |  | R07E5.7 |  |
|  |  |  |  |  |  |  |  |  |  |  |  |  |  |  |  |  |  |  |  |  |  |  |  |  |  |  |  |  |  |  |  |  |  |  |  |  |  | *pqn-96* | Prion-like-(Q/N-rich)-domain-bearing protein |
|  |  |  |  |  |  |  |  |  |  |  |  |  |  |  |  |  |  |  |  |  |  |  |  |  |  |  |  |  |  |  |  |  |  |  |  |  |  | Y41C4A.8 |  |
|  |  |  |  |  |  |  |  |  |  |  |  |  |  |  |  |  |  |  |  |  |  |  |  |  |  |  |  |  |  |  |  |  |  |  |  |  |  | C25E10.8 |  |
|  |  |  |  |  |  |  |  |  |  |  |  |  |  |  |  |  |  |  |  |  |  |  |  |  |  |  |  |  |  |  |  |  |  |  |  |  |  | M01E5.4 |  |
|  |  |  |  |  |  |  |  |  |  |  |  |  |  |  |  |  |  |  |  |  |  |  |  |  |  |  |  |  |  |  |  |  |  |  |  |  |  | T28D6.7 |  |
|  |  |  |  |  |  |  |  |  |  |  |  |  |  |  |  |  |  |  |  |  |  |  |  |  |  |  |  |  |  |  |  |  |  |  |  |  |  | *cnp-2* | CalciNeurin binding Protein |
|  |  |  |  |  |  |  |  |  |  |  |  |  |  |  |  |  |  |  |  |  |  |  |  |  |  |  |  |  |  |  |  |  |  |  |  |  |  | F42F12.3 |  |
|  |  |  |  |  |  |  |  |  |  |  |  |  |  |  |  |  |  |  |  |  |  |  |  |  |  |  |  |  |  |  |  |  |  |  |  |  |  | H16D19.5 |  |
|  |  |  |  |  |  |  |  |  |  |  |  |  |  |  |  |  |  |  |  |  |  |  |  |  |  |  |  |  |  |  |  |  |  |  |  |  |  | *str-18* | Seven TM Receptor |
|  |  |  |  |  |  |  |  |  |  |  |  |  |  |  |  |  |  |  |  |  |  |  |  |  |  |  |  |  |  |  |  |  |  |  |  |  |  | R12C12.5 |  |
|  |  |  |  |  |  |  |  |  |  |  |  |  |  |  |  |  |  |  |  |  |  |  |  |  |  |  |  |  |  |  |  |  |  |  |  |  |  | *cutl-22* | CUTiclin-Like |
|  |  |  |  |  |  |  |  |  |  |  |  |  |  |  |  |  |  |  |  |  |  |  |  |  |  |  |  |  |  |  |  |  |  |  |  |  |  | *ugt-28* | UDP-GlucuronosylTransferase |
|  |  |  |  |  |  |  |  |  |  |  |  |  |  |  |  |  |  |  |  |  |  |  |  |  |  |  |  |  |  |  |  |  |  |  |  |  |  | C07E3.3 |  |
|  |  |  |  |  |  |  |  |  |  |  |  |  |  |  |  |  |  |  |  |  |  |  |  |  |  |  |  |  |  |  |  |  |  |  |  |  |  | W05B10.4 |  |
|  |  |  |  |  |  |  |  |  |  |  |  |  |  |  |  |  |  |  |  |  |  |  |  |  |  |  |  |  |  |  |  |  |  |  |  |  |  | *eff-1* | Epithelial Fusion Failure |
|  |  |  |  |  |  |  |  |  |  |  |  |  |  |  |  |  |  |  |  |  |  |  |  |  |  |  |  |  |  |  |  |  |  |  |  |  |  | C32D5.1 |  |
|  |  |  |  |  |  |  |  |  |  |  |  |  |  |  |  |  |  |  |  |  |  |  |  |  |  |  |  |  |  |  |  |  |  |  |  |  |  | T23G5.3 |  |
|  |  |  |  |  |  |  |  |  |  |  |  |  |  |  |  |  |  |  |  |  |  |  |  |  |  |  |  |  |  |  |  |  |  |  |  |  |  | *srp-6* | SeRPin |
|  |  |  |  |  |  |  |  |  |  |  |  |  |  |  |  |  |  |  |  |  |  |  |  |  |  |  |  |  |  |  |  |  |  |  |  |  |  | F47B8.2 |  |
|  |  |  |  |  |  |  |  |  |  |  |  |  |  |  |  |  |  |  |  |  |  |  |  |  |  |  |  |  |  |  |  |  |  |  |  |  |  | T15B12.1 |  |
|  |  |  |  |  |  |  |  |  |  |  |  |  |  |  |  |  |  |  |  |  |  |  |  |  |  |  |  |  |  |  |  |  |  |  |  |  |  | *nhr-18* | Nuclear Hormone Receptor family |
|  |  |  |  |  |  |  |  |  |  |  |  |  |  |  |  |  |  |  |  |  |  |  |  |  |  |  |  |  |  |  |  |  |  |  |  |  |  | *nhr-102* | Nuclear Hormone Receptor family |
|  |  |  |  |  |  |  |  |  |  |  |  |  |  |  |  |  |  |  |  |  |  |  |  |  |  |  |  |  |  |  |  |  |  |  |  |  |  | *nhr-184* | Nuclear Hormone Receptor family |
|  |  |  |  |  |  |  |  |  |  |  |  |  |  |  |  |  |  |  |  |  |  |  |  |  |  |  |  |  |  |  |  |  |  |  |  |  |  | *nhr-134* | Nuclear Hormone Receptor family |
|  |  |  |  |  |  |  |  |  |  |  |  |  |  |  |  |  |  |  |  |  |  |  |  |  |  |  |  |  |  |  |  |  |  |  |  |  |  | *nhr-96* | Nuclear Hormone Receptor family |
|  |  |  |  |  |  |  |  |  |  |  |  |  |  |  |  |  |  |  |  |  |  |  |  |  |  |  |  |  |  |  |  |  |  |  |  |  |  | T26C5.4 |  |
|  |  |  |  |  |  |  |  |  |  |  |  |  |  |  |  |  |  |  |  |  |  |  |  |  |  |  |  |  |  |  |  |  |  |  |  |  |  | *grl-14* | GRound-Like (grd related) |
|  |  |  |  |  |  |  |  |  |  |  |  |  |  |  |  |  |  |  |  |  |  |  |  |  |  |  |  |  |  |  |  |  |  |  |  |  |  | F47E1.3 |  |
|  |  |  |  |  |  |  |  |  |  |  |  |  |  |  |  |  |  |  |  |  |  |  |  |  |  |  |  |  |  |  |  |  |  |  |  |  |  | *cpn-1* | CalPoNin |
|  |  |  |  |  |  |  |  |  |  |  |  |  |  |  |  |  |  |  |  |  |  |  |  |  |  |  |  |  |  |  |  |  |  |  |  |  |  | F35D2.6 |  |
|  |  |  |  |  |  |  |  |  |  |  |  |  |  |  |  |  |  |  |  |  |  |  |  |  |  |  |  |  |  |  |  |  |  |  |  |  |  | *sssh-1* | SleepleSS (Drosophila) Homolog |
|  |  |  |  |  |  |  |  |  |  |  |  |  |  |  |  |  |  |  |  |  |  |  |  |  |  |  |  |  |  |  |  |  |  |  |  |  |  | F25H5.7 |  |
|  |  |  |  |  |  |  |  |  |  |  |  |  |  |  |  |  |  |  |  |  |  |  |  |  |  |  |  |  |  |  |  |  |  |  |  |  |  | *spp-22* | SaPosin-like Protein family |
|  |  |  |  |  |  |  |  |  |  |  |  |  |  |  |  |  |  |  |  |  |  |  |  |  |  |  |  |  |  |  |  |  |  |  |  |  |  | *egl-6* | EGg Laying defective |
|  |  |  |  |  |  |  |  |  |  |  |  |  |  |  |  |  |  |  |  |  |  |  |  |  |  |  |  |  |  |  |  |  |  |  |  |  |  | *srxa-8* | Serpentine Receptor, class XA |
|  |  |  |  |  |  |  |  |  |  |  |  |  |  |  |  |  |  |  |  |  |  |  |  |  |  |  |  |  |  |  |  |  |  |  |  |  |  | D2024.4 |  |
|  |  |  |  |  |  |  |  |  |  |  |  |  |  |  |  |  |  |  |  |  |  |  |  |  |  |  |  |  |  |  |  |  |  |  |  |  |  | R07B1.8 |  |
|  |  |  |  |  |  |  |  |  |  |  |  |  |  |  |  |  |  |  |  |  |  |  |  |  |  |  |  |  |  |  |  |  |  |  |  |  |  | F45E4.5 |  |
|  |  |  |  |  |  |  |  |  |  |  |  |  |  |  |  |  |  |  |  |  |  |  |  |  |  |  |  |  |  |  |  |  |  |  |  |  |  | F35A5.5 |  |
|  |  |  |  |  |  |  |  |  |  |  |  |  |  |  |  |  |  |  |  |  |  |  |  |  |  |  |  |  |  |  |  |  |  |  |  |  |  | *lagr-1* | LAG1 (yeast Longevity-Assurance Gene) Related |
|  |  |  |  |  |  |  |  |  |  |  |  |  |  |  |  |  |  |  |  |  |  |  |  |  |  |  |  |  |  |  |  |  |  |  |  |  |  | T27E4.5 |  |
|  |  |  |  |  |  |  |  |  |  |  |  |  |  |  |  |  |  |  |  |  |  |  |  |  |  |  |  |  |  |  |  |  |  |  |  |  |  | C16D9.1 |  |
|  |  |  |  |  |  |  |  |  |  |  |  |  |  |  |  |  |  |  |  |  |  |  |  |  |  |  |  |  |  |  |  |  |  |  |  |  |  | K08D12.8 |  |
|  |  |  |  |  |  |  |  |  |  |  |  |  |  |  |  |  |  |  |  |  |  |  |  |  |  |  |  |  |  |  |  |  |  |  |  |  |  | ZK662.2 |  |
|  |  |  |  |  |  |  |  |  |  |  |  |  |  |  |  |  |  |  |  |  |  |  |  |  |  |  |  |  |  |  |  |  |  |  |  |  |  | *grh-1* | GRainyHead (Drosophila transcription factor) homolog |
|  |  |  |  |  |  |  |  |  |  |  |  |  |  |  |  |  |  |  |  |  |  |  |  |  |  |  |  |  |  |  |  |  |  |  |  |  |  | F57G12.2 |  |
|  |  |  |  |  |  |  |  |  |  |  |  |  |  |  |  |  |  |  |  |  |  |  |  |  |  |  |  |  |  |  |  |  |  |  |  |  |  | T07F12.1 |  |
|  |  |  |  |  |  |  |  |  |  |  |  |  |  |  |  |  |  |  |  |  |  |  |  |  |  |  |  |  |  |  |  |  |  |  |  |  |  | T28C6.8 |  |
|  |  |  |  |  |  |  |  |  |  |  |  |  |  |  |  |  |  |  |  |  |  |  |  |  |  |  |  |  |  |  |  |  |  |  |  |  |  | C50B6.9 |  |
|  |  |  |  |  |  |  |  |  |  |  |  |  |  |  |  |  |  |  |  |  |  |  |  |  |  |  |  |  |  |  |  |  |  |  |  |  |  | Y22D7AR.6 |  |
|  |  |  |  |  |  |  |  |  |  |  |  |  |  |  |  |  |  |  |  |  |  |  |  |  |  |  |  |  |  |  |  |  |  |  |  |  |  | *nhr-146* | Nuclear Hormone Receptor family |
|  |  |  |  |  |  |  |  |  |  |  |  |  |  |  |  |  |  |  |  |  |  |  |  |  |  |  |  |  |  |  |  |  |  |  |  |  |  | *nhr-204* | Nuclear Hormone Receptor family |
|  |  |  |  |  |  |  |  |  |  |  |  |  |  |  |  |  |  |  |  |  |  |  |  |  |  |  |  |  |  |  |  |  |  |  |  |  |  | *ztf-27* | Zinc finger putative Transcription Factor family |
|  |  |  |  |  |  |  |  |  |  |  |  |  |  |  |  |  |  |  |  |  |  |  |  |  |  |  |  |  |  |  |  |  |  |  |  |  |  | *nhr-70* | Nuclear Hormone Receptor family |
|  |  |  |  |  |  |  |  |  |  |  |  |  |  |  |  |  |  |  |  |  |  |  |  |  |  |  |  |  |  |  |  |  |  |  |  |  |  | Y47G6A.18 |  |
|  |  |  |  |  |  |  |  |  |  |  |  |  |  |  |  |  |  |  |  |  |  |  |  |  |  |  |  |  |  |  |  |  |  |  |  |  |  | *vab-9* | Variable ABnormal morphology |
|  |  |  |  |  |  |  |  |  |  |  |  |  |  |  |  |  |  |  |  |  |  |  |  |  |  |  |  |  |  |  |  |  |  |  |  |  |  | *mca-2* | Membrane Calcium ATPase |
|  |  |  |  |  |  |  |  |  |  |  |  |  |  |  |  |  |  |  |  |  |  |  |  |  |  |  |  |  |  |  |  |  |  |  |  |  |  | F36H2.5 |  |
|  |  |  |  |  |  |  |  |  |  |  |  |  |  |  |  |  |  |  |  |  |  |  |  |  |  |  |  |  |  |  |  |  |  |  |  |  |  | T05D4.3 |  |
|  |  |  |  |  |  |  |  |  |  |  |  |  |  |  |  |  |  |  |  |  |  |  |  |  |  |  |  |  |  |  |  |  |  |  |  |  |  | *ptr-11* | PaTched Related family |
|  |  |  |  |  |  |  |  |  |  |  |  |  |  |  |  |  |  |  |  |  |  |  |  |  |  |  |  |  |  |  |  |  |  |  |  |  |  | *ifb-1* | Intermediate Filament, B |
|  |  |  |  |  |  |  |  |  |  |  |  |  |  |  |  |  |  |  |  |  |  |  |  |  |  |  |  |  |  |  |  |  |  |  |  |  |  | *clp-7* | CaLPain family |
|  |  |  |  |  |  |  |  |  |  |  |  |  |  |  |  |  |  |  |  |  |  |  |  |  |  |  |  |  |  |  |  |  |  |  |  |  |  | *pat-12* | Paralysed Arrest at Two-fold |
|  |  |  |  |  |  |  |  |  |  |  |  |  |  |  |  |  |  |  |  |  |  |  |  |  |  |  |  |  |  |  |  |  |  |  |  |  |  | *brp-1* | Bypass of Response to Pheromone in yeast |
|  |  |  |  |  |  |  |  |  |  |  |  |  |  |  |  |  |  |  |  |  |  |  |  |  |  |  |  |  |  |  |  |  |  |  |  |  |  | *rhgf-2* | RHo Guanine nucleotide exchange Factor |
|  |  |  |  |  |  |  |  |  |  |  |  |  |  |  |  |  |  |  |  |  |  |  |  |  |  |  |  |  |  |  |  |  |  |  |  |  |  | *daf-12* | abnormal DAuer Formation |
|  |  |  |  |  |  |  |  |  |  |  |  |  |  |  |  |  |  |  |  |  |  |  |  |  |  |  |  |  |  |  |  |  |  |  |  |  |  | C25A1.5 |  |
|  |  |  |  |  |  |  |  |  |  |  |  |  |  |  |  |  |  |  |  |  |  |  |  |  |  |  |  |  |  |  |  |  |  |  |  |  |  | *nkb-3* | Na+/K+ ATPase, Beta subunit |
|  |  |  |  |  |  |  |  |  |  |  |  |  |  |  |  |  |  |  |  |  |  |  |  |  |  |  |  |  |  |  |  |  |  |  |  |  |  | *dhhc-2* | DHHC-types zinc finger protein |
|  |  |  |  |  |  |  |  |  |  |  |  |  |  |  |  |  |  |  |  |  |  |  |  |  |  |  |  |  |  |  |  |  |  |  |  |  |  | C24G6.2 |  |
|  |  |  |  |  |  |  |  |  |  |  |  |  |  |  |  |  |  |  |  |  |  |  |  |  |  |  |  |  |  |  |  |  |  |  |  |  |  | *fkh-9* | ForKHead transcription factor family |
|  |  |  |  |  |  |  |  |  |  |  |  |  |  |  |  |  |  |  |  |  |  |  |  |  |  |  |  |  |  |  |  |  |  |  |  |  |  | C14A4.12 |  |
|  |  |  |  |  |  |  |  |  |  |  |  |  |  |  |  |  |  |  |  |  |  |  |  |  |  |  |  |  |  |  |  |  |  |  |  |  |  | R08C7.12 |  |
|  |  |  |  |  |  |  |  |  |  |  |  |  |  |  |  |  |  |  |  |  |  |  |  |  |  |  |  |  |  |  |  |  |  |  |  |  |  | *hhat-2* | Homolog of Hedgehog AcylTransferase |
|  |  |  |  |  |  |  |  |  |  |  |  |  |  |  |  |  |  |  |  |  |  |  |  |  |  |  |  |  |  |  |  |  |  |  |  |  |  | F59F5.7 |  |
|  |  |  |  |  |  |  |  |  |  |  |  |  |  |  |  |  |  |  |  |  |  |  |  |  |  |  |  |  |  |  |  |  |  |  |  |  |  | K02G10.5 |  |
|  |  |  |  |  |  |  |  |  |  |  |  |  |  |  |  |  |  |  |  |  |  |  |  |  |  |  |  |  |  |  |  |  |  |  |  |  |  | R11F4.3 |  |
|  |  |  |  |  |  |  |  |  |  |  |  |  |  |  |  |  |  |  |  |  |  |  |  |  |  |  |  |  |  |  |  |  |  |  |  |  |  | *daf-6* | abnormal DAuer Formation |
|  |  |  |  |  |  |  |  |  |  |  |  |  |  |  |  |  |  |  |  |  |  |  |  |  |  |  |  |  |  |  |  |  |  |  |  |  |  | *che-14* | abnormal CHEmotaxis |
|  |  |  |  |  |  |  |  |  |  |  |  |  |  |  |  |  |  |  |  |  |  |  |  |  |  |  |  |  |  |  |  |  |  |  |  |  |  | *blmp-1* | Blimp1 (B Lymphocyte-induced Maturation Protein-1) homolog |
|  |  |  |  |  |  |  |  |  |  |  |  |  |  |  |  |  |  |  |  |  |  |  |  |  |  |  |  |  |  |  |  |  |  |  |  |  |  | C01C10.2 |  |
|  |  |  |  |  |  |  |  |  |  |  |  |  |  |  |  |  |  |  |  |  |  |  |  |  |  |  |  |  |  |  |  |  |  |  |  |  |  | D2092.8 |  |
|  |  |  |  |  |  |  |  |  |  |  |  |  |  |  |  |  |  |  |  |  |  |  |  |  |  |  |  |  |  |  |  |  |  |  |  |  |  | T19C3.2 |  |
|  |  |  |  |  |  |  |  |  |  |  |  |  |  |  |  |  |  |  |  |  |  |  |  |  |  |  |  |  |  |  |  |  |  |  |  |  |  | *tpst-1* | TyrosylProtein SulfoTransferase |
|  |  |  |  |  |  |  |  |  |  |  |  |  |  |  |  |  |  |  |  |  |  |  |  |  |  |  |  |  |  |  |  |  |  |  |  |  |  | ZC190.4 |  |
|  |  |  |  |  |  |  |  |  |  |  |  |  |  |  |  |  |  |  |  |  |  |  |  |  |  |  |  |  |  |  |  |  |  |  |  |  |  | *nhr-34* | Nuclear Hormone Receptor family |
|  |  |  |  |  |  |  |  |  |  |  |  |  |  |  |  |  |  |  |  |  |  |  |  |  |  |  |  |  |  |  |  |  |  |  |  |  |  | *spin-1* | SPINster (Drosophila lysosomal permease) homolog |
|  |  |  |  |  |  |  |  |  |  |  |  |  |  |  |  |  |  |  |  |  |  |  |  |  |  |  |  |  |  |  |  |  |  |  |  |  |  | *fln-2* | FiLamiN (actin binding protein) homolog |
|  |  |  |  |  |  |  |  |  |  |  |  |  |  |  |  |  |  |  |  |  |  |  |  |  |  |  |  |  |  |  |  |  |  |  |  |  |  | *cal-5* | CALmodulin related genes |
|  |  |  |  |  |  |  |  |  |  |  |  |  |  |  |  |  |  |  |  |  |  |  |  |  |  |  |  |  |  |  |  |  |  |  |  |  |  | *dkf-1* | D Kinase Family |
|  |  |  |  |  |  |  |  |  |  |  |  |  |  |  |  |  |  |  |  |  |  |  |  |  |  |  |  |  |  |  |  |  |  |  |  |  |  | C06A6.5 |  |
|  |  |  |  |  |  |  |  |  |  |  |  |  |  |  |  |  |  |  |  |  |  |  |  |  |  |  |  |  |  |  |  |  |  |  |  |  |  | *ddr-1* | Discoidin Domain Receptor |
|  |  |  |  |  |  |  |  |  |  |  |  |  |  |  |  |  |  |  |  |  |  |  |  |  |  |  |  |  |  |  |  |  |  |  |  |  |  | *vab-10* | Variable ABnormal morphology |
|  |  |  |  |  |  |  |  |  |  |  |  |  |  |  |  |  |  |  |  |  |  |  |  |  |  |  |  |  |  |  |  |  |  |  |  |  |  | F07C6.4 |  |
|  |  |  |  |  |  |  |  |  |  |  |  |  |  |  |  |  |  |  |  |  |  |  |  |  |  |  |  |  |  |  |  |  |  |  |  |  |  | *cutl-3* | CUTiclin-Like |
|  |  |  |  |  |  |  |  |  |  |  |  |  |  |  |  |  |  |  |  |  |  |  |  |  |  |  |  |  |  |  |  |  |  |  |  |  |  | *sym-2* | SYnthetic lethal with Mec |
|  |  |  |  |  |  |  |  |  |  |  |  |  |  |  |  |  |  |  |  |  |  |  |  |  |  |  |  |  |  |  |  |  |  |  |  |  |  | *gly-11* | GLYcosylation related |
|  |  |  |  |  |  |  |  |  |  |  |  |  |  |  |  |  |  |  |  |  |  |  |  |  |  |  |  |  |  |  |  |  |  |  |  |  |  | *exc-6* | INverted Formin/formin Three-related |
|  |  |  |  |  |  |  |  |  |  |  |  |  |  |  |  |  |  |  |  |  |  |  |  |  |  |  |  |  |  |  |  |  |  |  |  |  |  | Y54G2A.4 |  |
|  |  |  |  |  |  |  |  |  |  |  |  |  |  |  |  |  |  |  |  |  |  |  |  |  |  |  |  |  |  |  |  |  |  |  |  |  |  | Y65A5A.1 |  |
|  |  |  |  |  |  |  |  |  |  |  |  |  |  |  |  |  |  |  |  |  |  |  |  |  |  |  |  |  |  |  |  |  |  |  |  |  |  | K10B4.3 |  |
|  |  |  |  |  |  |  |  |  |  |  |  |  |  |  |  |  |  |  |  |  |  |  |  |  |  |  |  |  |  |  |  |  |  |  |  |  |  | *nex-2* | anNEXin family |
|  |  |  |  |  |  |  |  |  |  |  |  |  |  |  |  |  |  |  |  |  |  |  |  |  |  |  |  |  |  |  |  |  |  |  |  |  |  | *egl-26* | EGg Laying defective |
|  |  |  |  |  |  |  |  |  |  |  |  |  |  |  |  |  |  |  |  |  |  |  |  |  |  |  |  |  |  |  |  |  |  |  |  |  |  | F59B2.2 |  |
|  |  |  |  |  |  |  |  |  |  |  |  |  |  |  |  |  |  |  |  |  |  |  |  |  |  |  |  |  |  |  |  |  |  |  |  |  |  | T26C12.3 |  |
|  |  |  |  |  |  |  |  |  |  |  |  |  |  |  |  |  |  |  |  |  |  |  |  |  |  |  |  |  |  |  |  |  |  |  |  |  |  | *ref-2* | REgulator of Fusion |
|  |  |  |  |  |  |  |  |  |  |  |  |  |  |  |  |  |  |  |  |  |  |  |  |  |  |  |  |  |  |  |  |  |  |  |  |  |  | *bam-2* | Branching AbnorMal |
|  |  |  |  |  |  |  |  |  |  |  |  |  |  |  |  |  |  |  |  |  |  |  |  |  |  |  |  |  |  |  |  |  |  |  |  |  |  | F53A9.7 |  |
|  |  |  |  |  |  |  |  |  |  |  |  |  |  |  |  |  |  |  |  |  |  |  |  |  |  |  |  |  |  |  |  |  |  |  |  |  |  | Y43H11AL.5 |  |
|  |  |  |  |  |  |  |  |  |  |  |  |  |  |  |  |  |  |  |  |  |  |  |  |  |  |  |  |  |  |  |  |  |  |  |  |  |  | F57A10.8 |  |
|  |  |  |  |  |  |  |  |  |  |  |  |  |  |  |  |  |  |  |  |  |  |  |  |  |  |  |  |  |  |  |  |  |  |  |  |  |  | *gst-19* | Glutathione S-Transferase |
|  |  |  |  |  |  |  |  |  |  |  |  |  |  |  |  |  |  |  |  |  |  |  |  |  |  |  |  |  |  |  |  |  |  |  |  |  |  | K08D10.9 |  |
|  |  |  |  |  |  |  |  |  |  |  |  |  |  |  |  |  |  |  |  |  |  |  |  |  |  |  |  |  |  |  |  |  |  |  |  |  |  | F14F9.6 |  |
|  |  |  |  |  |  |  |  |  |  |  |  |  |  |  |  |  |  |  |  |  |  |  |  |  |  |  |  |  |  |  |  |  |  |  |  |  |  | Y105E8A.48 |  |
|  |  |  |  |  |  |  |  |  |  |  |  |  |  |  |  |  |  |  |  |  |  |  |  |  |  |  |  |  |  |  |  |  |  |  |  |  |  | F58G1.12 |  |
|  |  |  |  |  |  |  |  |  |  |  |  |  |  |  |  |  |  |  |  |  |  |  |  |  |  |  |  |  |  |  |  |  |  |  |  |  |  | T08H4.6 |  |
|  |  |  |  |  |  |  |  |  |  |  |  |  |  |  |  |  |  |  |  |  |  |  |  |  |  |  |  |  |  |  |  |  |  |  |  |  |  | *fipr-1* | FIP (Fungus-Induced Protein) Related |
|  |  |  |  |  |  |  |  |  |  |  |  |  |  |  |  |  |  |  |  |  |  |  |  |  |  |  |  |  |  |  |  |  |  |  |  |  |  | F11G11.5 |  |
|  |  |  |  |  |  |  |  |  |  |  |  |  |  |  |  |  |  |  |  |  |  |  |  |  |  |  |  |  |  |  |  |  |  |  |  |  |  | *glt-5* | GLutamate Transporter family |
|  |  |  |  |  |  |  |  |  |  |  |  |  |  |  |  |  |  |  |  |  |  |  |  |  |  |  |  |  |  |  |  |  |  |  |  |  |  | F28H7.3 |  |
|  |  |  |  |  |  |  |  |  |  |  |  |  |  |  |  |  |  |  |  |  |  |  |  |  |  |  |  |  |  |  |  |  |  |  |  |  |  | C36B7.4 |  |
|  |  |  |  |  |  |  |  |  |  |  |  |  |  |  |  |  |  |  |  |  |  |  |  |  |  |  |  |  |  |  |  |  |  |  |  |  |  | *nhl-3* | NHL (ring finger b-box coiled coil) domain containing |
|  |  |  |  |  |  |  |  |  |  |  |  |  |  |  |  |  |  |  |  |  |  |  |  |  |  |  |  |  |  |  |  |  |  |  |  |  |  | *hlh-30* | Helix Loop Helix |
|  |  |  |  |  |  |  |  |  |  |  |  |  |  |  |  |  |  |  |  |  |  |  |  |  |  |  |  |  |  |  |  |  |  |  |  |  |  | *cogc-6* | Conserved Oligomeric Golgi (COG) Component |
|  |  |  |  |  |  |  |  |  |  |  |  |  |  |  |  |  |  |  |  |  |  |  |  |  |  |  |  |  |  |  |  |  |  |  |  |  |  | *mig-23* | abnormal cell MIGration |
|  |  |  |  |  |  |  |  |  |  |  |  |  |  |  |  |  |  |  |  |  |  |  |  |  |  |  |  |  |  |  |  |  |  |  |  |  |  | Y44E3A.4 |  |
|  |  |  |  |  |  |  |  |  |  |  |  |  |  |  |  |  |  |  |  |  |  |  |  |  |  |  |  |  |  |  |  |  |  |  |  |  |  | *tat-5* | Transbilayer Amphipath Transporters (subfamily IV P-type ATPase) |
|  |  |  |  |  |  |  |  |  |  |  |  |  |  |  |  |  |  |  |  |  |  |  |  |  |  |  |  |  |  |  |  |  |  |  |  |  |  | *gly-3* | GLYcosylation related |
|  |  |  |  |  |  |  |  |  |  |  |  |  |  |  |  |  |  |  |  |  |  |  |  |  |  |  |  |  |  |  |  |  |  |  |  |  |  | F38A1.8 |  |
|  |  |  |  |  |  |  |  |  |  |  |  |  |  |  |  |  |  |  |  |  |  |  |  |  |  |  |  |  |  |  |  |  |  |  |  |  |  | *vacl-14* | VAC (yeast VACuole morphology)-Like |
|  |  |  |  |  |  |  |  |  |  |  |  |  |  |  |  |  |  |  |  |  |  |  |  |  |  |  |  |  |  |  |  |  |  |  |  |  |  | T26C5.3 |  |
|  |  |  |  |  |  |  |  |  |  |  |  |  |  |  |  |  |  |  |  |  |  |  |  |  |  |  |  |  |  |  |  |  |  |  |  |  |  | *cgp-1* | Caenorhabditis GTP-binding Protein |
|  |  |  |  |  |  |  |  |  |  |  |  |  |  |  |  |  |  |  |  |  |  |  |  |  |  |  |  |  |  |  |  |  |  |  |  |  |  | *dnj-20* | DNaJ domain (prokaryotic heat shock protein) |
|  |  |  |  |  |  |  |  |  |  |  |  |  |  |  |  |  |  |  |  |  |  |  |  |  |  |  |  |  |  |  |  |  |  |  |  |  |  | *tfg-1* |  |
|  |  |  |  |  |  |  |  |  |  |  |  |  |  |  |  |  |  |  |  |  |  |  |  |  |  |  |  |  |  |  |  |  |  |  |  |  |  | R148.3 |  |
|  |  |  |  |  |  |  |  |  |  |  |  |  |  |  |  |  |  |  |  |  |  |  |  |  |  |  |  |  |  |  |  |  |  |  |  |  |  | *frm-5.2* | FERM domain (protein4.1-ezrin-radixin-moesin) family |
|  |  |  |  |  |  |  |  |  |  |  |  |  |  |  |  |  |  |  |  |  |  |  |  |  |  |  |  |  |  |  |  |  |  |  |  |  |  | Y42H9AR.1 |  |
|  |  |  |  |  |  |  |  |  |  |  |  |  |  |  |  |  |  |  |  |  |  |  |  |  |  |  |  |  |  |  |  |  |  |  |  |  |  | K08C9.8 |  |
|  |  |  |  |  |  |  |  |  |  |  |  |  |  |  |  |  |  |  |  |  |  |  |  |  |  |  |  |  |  |  |  |  |  |  |  |  |  | F53A3.5 |  |
|  |  |  |  |  |  |  |  |  |  |  |  |  |  |  |  |  |  |  |  |  |  |  |  |  |  |  |  |  |  |  |  |  |  |  |  |  |  | C29G2.2 |  |
|  |  |  |  |  |  |  |  |  |  |  |  |  |  |  |  |  |  |  |  |  |  |  |  |  |  |  |  |  |  |  |  |  |  |  |  |  |  | C25F6.19 |  |
|  |  |  |  |  |  |  |  |  |  |  |  |  |  |  |  |  |  |  |  |  |  |  |  |  |  |  |  |  |  |  |  |  |  |  |  |  |  | C25F6.22 |  |
|  |  |  |  |  |  |  |  |  |  |  |  |  |  |  |  |  |  |  |  |  |  |  |  |  |  |  |  |  |  |  |  |  |  |  |  |  |  | *chat-1* | CHAperonin of TAT-1 |
|  |  |  |  |  |  |  |  |  |  |  |  |  |  |  |  |  |  |  |  |  |  |  |  |  |  |  |  |  |  |  |  |  |  |  |  |  |  | Y75B8A.59 |  |
|  |  |  |  |  |  |  |  |  |  |  |  |  |  |  |  |  |  |  |  |  |  |  |  |  |  |  |  |  |  |  |  |  |  |  |  |  |  | *frm-5.1* | FERM domain (protein4.1-ezrin-radixin-moesin) family |
|  |  |  |  |  |  |  |  |  |  |  |  |  |  |  |  |  |  |  |  |  |  |  |  |  |  |  |  |  |  |  |  |  |  |  |  |  |  | K12H4.t1 |  |
|  |  |  |  |  |  |  |  |  |  |  |  |  |  |  |  |  |  |  |  |  |  |  |  |  |  |  |  |  |  |  |  |  |  |  |  |  |  | C16B8.13 |  |
|  |  |  |  |  |  |  |  |  |  |  |  |  |  |  |  |  |  |  |  |  |  |  |  |  |  |  |  |  |  |  |  |  |  |  |  |  |  | C26B9.1 |  |

### Phenotypes enriched

none found

### Anatomy terms enriched

none found

### GO terms enriched

|  |  |  |
| --- | --- | --- |
| **GO term** | **Number of genes** | **FDR-corrected p-value** |
| cellular response to hormone stimulus | 13 | 0.0026 |
| steroid hormone receptor activity | 12 | 0.0042 |
| response to endogenous stimulus | 13 | 0.0059 |
| steroid hormone mediated signaling pathway | 12 | 0.0059 |
| response to steroid hormone | 12 | 0.0059 |
| cellular response to lipid | 12 | 0.0059 |
| cellular response to organic cyclic compound | 12 | 0.0075 |
| response to organic substance | 13 | 0.0170 |
| cellular response to chemical stimulus | 13 | 0.0190 |
| Golgi membrane | 6 | 0.0280 |

### Expression clusters enriched

|  |  |  |  |
| --- | --- | --- | --- |
| **Group name** | **Number in cluster** | **Enrichment** | **FDR corrected p** |
| Total muscle depleted genes (complete list of non-overlapping genes from the 0hr and 24hr muscle depleted datasets). | 67 | 2.30 | 5.46e-09 |
| Genes depleted in muscle cells (0hr muscle dataset). Dissociated myo-3::GFP embryos were cultured for 0 hours before FACS sorting. | 49 | 2.45 | 1.05e-06 |
| Genes depleted in muscle cells (24hr muscle dataset). Dissociated myo-3::GFP embryos were cultured for 24 hours before FACS sorting. | 44 | 2.36 | 2.08e-05 |
| Genes upregulated in dcr-1(-/-) adult animals by at least 1.5 fold and P < 0.01, as determined by a multisample t-test and the Benjamini and Hochberg false discovery rate correction. | 34 | 2.69 | 4.44e-05 |
| Genes differentially expressed in control vs under EtBr treatment without UVC exposure, at the -1h timepoint. | 77 | 1.53 | 4.26e-03 |
| Genes with significant variation across both genotypes and environments. Genotypes | 31 | 2.16 | 1.01e-02 |
| Genes that show selective expression in a subset of cell types vs broadly expressed in many cell types. Correspond to 20% - 57% of enriched\_genes for a given cell type. WBPaper00037950:excretory-cell\_larva\_SelectivelyEnriched | 12 | 4.11 | 1.03e-02 |
| Genes that show selective expression in a subset of cell types vs broadly expressed in many cell types. Correspond to 20% - 57% of enriched\_genes for a given cell type. WBPaper00037950:GABAergic-motor-neurons\_larva\_SelectivelyEnriched | 12 | 4.11 | 1.03e-02 |
| Genes upregulated by alg-1(-). | 34 | 2.04 | 1.23e-02 |
| Genes up regulated by mir-243(n4759). | 39 | 1.85 | 2.49e-02 |
| Significantly upregulated genes from cyc-1(RNAi) microarrays using SAM algorithm with an FDR < 0.1 from adult-only chips. | 67 | 1.51 | 2.83e-02 |
| Genes with no change in hcf-1(-), no change in sir-2.1(O/E) and upregulated in daf-2(-). | 24 | 2.26 | 3.60e-02 |
| Genes with variation in expression level across genotypes (AB2, CB4856, RC301, CB4857, N2, HC445). | 41 | 1.77 | 3.75e-02 |
| Germline-enriched and sex-biased expression profile cluster B. | 21 | 2.38 | 4.46e-02 |

### Motifs enriched

|  |  |  |  |  |  |
| --- | --- | --- | --- | --- | --- |
| **Motif** | **Logo** | **Possible orthologs** | **Number of motifs in cluster** | **Enrichment** | **FDR corrected p** |
| MA0446.1 |  | lin-31 let-381 | 100 | 1.89 | 5.6e-10 |
| CG2052\_SOLEXA\_2.5\_FBgn0039905 |  | lin-29 (0.68) fkh-7 (0.66) mel-28 | 161 | 1.34 | 5.6e-09 |
| SP1\_f2 |  | klf-2 (0.74) ZC328.2 (0.52) klf-1 | 98 | 1.77 | 4.4e-08 |
| FOXJ2\_3 |  | pha-4 (0.76) fkh-7 (0.66) lin-31 let-381 fkh-8 fkh-10 daf-16 | 133 | 1.47 | 1.3e-07 |
| FOXO1\_3 |  | ZC328.2 (0.52) daf-16 | 109 | 1.64 | 1.3e-07 |
| Sox8\_1733 |  | gei-3 sox-4 pop-1 C05C9.3 | 132 | 1.47 | 1.5e-07 |
| PURA\_f1 |  | klf-2 (0.74) Y53H1A.2 (-0.66) plp-2 | 99 | 1.72 | 1.7e-07 |
| pTH9237 |  | mel-28 | 128 | 1.48 | 3.1e-07 |
| MA0543.1 |  | daf-8 eor-1 | 141 | 1.40 | 3.3e-07 |
| pTH10797 |  | lin-29 (0.68) K11D2.4 | 158 | 1.30 | 4.3e-07 |
| SRP000712\_Sox2 |  | ceh-18 ceh-6 sox-4 tbp-1 | 149 | 1.34 | 9.1e-07 |
| CG8765\_SANGER\_5\_FBgn0036900 |  | H20J04.3 | 128 | 1.46 | 9.7e-07 |
| pTH10624 |  | mef-2 (0.66) let-381 mel-28 Y61A9LA.9 Y116A8C.22 | 136 | 1.40 | 1.4e-06 |
| pTH3796 |  | lin-31 let-381 fkh-10 C34D1.1 | 103 | 1.61 | 1.6e-06 |
| MSX2\_f1 |  | eyg-1 (0.64) ceh-45 (0.55) ceh-1 lin-39 ceh-10 alr-1 | 124 | 1.47 | 1.7e-06 |
| Blimp-1\_SANGER\_5\_FBgn0035625 |  | blmp-1 (0.55) | 148 | 1.34 | 1.7e-06 |
| V$AREB6\_04 |  | ztf-6 gei-11 C34D1.1 | 88 | 1.74 | 1.7e-06 |
| POU4F2\_1 |  | unc-86 ceh-18 | 136 | 1.40 | 2.1e-06 |
| MA0135.1 |  | lin-39 unc-86 cfi-1 php-3 lim-7 | 116 | 1.51 | 2.2e-06 |
| pTH2846 |  | lin-31 | 97 | 1.65 | 2.3e-06 |
| Dll\_Cell\_FBgn0000157 |  | lin-39 ceh-18 ceh-12 ceh-43 alr-1 pal-1 | 124 | 1.46 | 2.6e-06 |
| nau\_da\_SANGER\_5\_FBgn0002922 |  | hlh-2 hlh-1 hlh-15 ces-1 K02D7.2 | 106 | 1.57 | 3.3e-06 |
| Foxa2\_2830 |  | fkh-7 (0.66) lin-31 let-381 fkh-8 fkh-10 daf-16 | 126 | 1.44 | 4.7e-06 |
| Plagl1\_0972 |  | Y53H1A.2 (-0.66) | 73 | 1.87 | 5.0e-06 |
| GRHL1\_2 |  | grh-1 (0.73) | 46 | 2.41 | 5.7e-06 |
| pTH9242 |  | mel-28 | 112 | 1.51 | 5.8e-06 |
| pTH1294 |  | mel-28 | 160 | 1.26 | 6.4e-06 |
| MA0249.1 |  | lin-31 hlh-32 hlh-8 | 104 | 1.56 | 6.6e-06 |
| pTH9355 |  | klf-2 (0.74) ZC328.2 (0.52) klf-1 | 101 | 1.58 | 7.1e-06 |
| PO3F2\_si |  | ceh-18 dmd-3 | 99 | 1.60 | 7.1e-06 |
| V$CEBP\_01 |  | C48E7.11 | 105 | 1.55 | 7.6e-06 |
| pTH9082 |  | mab-23 | 147 | 1.32 | 9.0e-06 |
| pTH6589 |  | nhr-213 (0.71) nhr-239 nhr-15 nhr-2 | 23 | 3.96 | 9.4e-06 |
| V$PBX1\_01 |  | ceh-20 lin-39 | 95 | 1.62 | 1.0e-05 |
| MA0016.1 |  | nhr-213 (0.71) nhr-10 nhr-19 nhr-2 nhr-69 | 82 | 1.73 | 1.0e-05 |
| SOX10\_1 |  | sox-4 egl-13 | 67 | 1.91 | 1.1e-05 |
| HLH26 |  | hlh-30 (0.67) mxl-1 (-0.57) ref-1 (0.54) mxl-2 lin-22 aha-1 hlh-26 C27D6.4 | 97 | 1.59 | 1.3e-05 |
| MA0143.3 |  | sox-4 pop-1 nhr-100 | 95 | 1.61 | 1.3e-05 |
| NR2F6\_f1 |  | nhr-62 (0.83) nhr-239 nhr-2 | 133 | 1.38 | 1.4e-05 |
| pTH3220 |  | daf-12 (0.8) Y5F2A.4 ceh-9 ztf-3 hif-1 | 89 | 1.65 | 1.5e-05 |
| rn\_SOLEXA\_5\_FBgn0259172 |  | lin-29 (0.68) | 152 | 1.28 | 1.5e-05 |
| KLF1\_f1 |  | klf-2 (0.74) klf-1 | 93 | 1.62 | 1.6e-05 |
| pTH8997 |  | hmg-12 (-0.76) ceh-53 (0.58) lin-39 lin-31 let-381 ceh-43 hmbx-1 Y116A8C.22 | 121 | 1.43 | 1.6e-05 |
| pTH9335 |  | mel-28 | 127 | 1.40 | 1.7e-05 |
| Mv90 |  | mef-2 (0.66) | 149 | 1.29 | 1.8e-05 |
| pTH9260 |  | mel-28 | 147 | 1.30 | 2.2e-05 |
| SOX2\_4 |  | grh-1 (0.73) sox-4 | 106 | 1.51 | 2.4e-05 |
| FOXO1\_si |  | fkh-9 (0.83) irx-1 daf-16 | 145 | 1.31 | 2.5e-05 |
| Sox15\_3457 |  | sox-4 | 104 | 1.52 | 2.5e-05 |
| pTH6497 |  | lin-31 | 118 | 1.44 | 2.6e-05 |
| pTH5667 |  | nhr-213 (0.71) nhr-71 (0.51) nhr-10 nhr-2 nhr-6 nhr-68 Y67D8A.3 | 38 | 2.54 | 3.0e-05 |
| NR2F6\_1 |  | nhr-19 nhr-2 | 86 | 1.65 | 3.4e-05 |
| HNF6\_f1 |  | ceh-48 (-0.54) dsc-1 | 123 | 1.40 | 3.5e-05 |
| Sp4\_1011 |  | klf-2 (0.74) sptf-3 klf-1 | 69 | 1.81 | 4.0e-05 |
| MA0049.1 |  | lin-39 php-3 hbl-1 | 149 | 1.28 | 4.1e-05 |
| MA0058.2 |  | hlh-30 (0.67) mxl-1 (-0.57) irx-1 | 91 | 1.60 | 4.3e-05 |
| HXD10\_f1 |  | nhr-2 php-3 | 136 | 1.34 | 4.4e-05 |
| pTH10042 |  | daf-12 (0.8) nhr-5 (0.59) | 94 | 1.57 | 4.6e-05 |
| HOXA10\_1 |  | ceh-13 lin-39 php-3 pal-1 ceh-24 T27F2.4 D1005.3 | 93 | 1.58 | 4.6e-05 |
| V$ZID\_01 |  | ztf-28 (-0.51) skn-1 ceh-32 | 105 | 1.49 | 5.1e-05 |
| HXD4\_f1 |  | ceh-19 (0.71) ceh-1 lin-39 cog-1 ceh-9 ceh-31 ceh-43 alr-1 ceh-24 lim-7 ceh-30 | 132 | 1.35 | 5.1e-05 |
| pTH10717 |  | syd-9 lsy-2 lsl-1 | 86 | 1.63 | 5.3e-05 |
| pTH10927 |  | mab-9 tbx-38 tbx-39 tbx-43 tbx-42 | 92 | 1.58 | 5.6e-05 |
| EVX2\_1 |  | ceh-2 (0.76) pha-2 (0.64) ceh-53 (0.58) lim-4 (-0.57) ceh-45 (0.55) lin-39 ceh-10 lim-6 cog-1 dsc-1 ceh-31 ceh-18 ceh-12 ceh-43 alr-1 mls-2 ceh-36 egl-5 pal-1 ceh-16 and 5 others  [full list] | 89 | 1.60 | 5.6e-05 |
| HXD13\_f1 |  | mex-6 pal-1 | 121 | 1.40 | 5.6e-05 |
| ARI3A\_do |  | nhr-208 (0.54) gei-3 cfi-1 | 133 | 1.35 | 5.9e-05 |
| pTH10779 |  | nhr-182 (0.79) nhr-134 (0.73) | 109 | 1.47 | 6.0e-05 |
| LHX2\_f1 |  | cfi-1 alr-1 ceh-14 ZC204.2 | 121 | 1.40 | 6.2e-05 |
| pTH9254 |  | mel-28 | 147 | 1.28 | 6.9e-05 |
| pTH1292 |  | pzf-1 ceh-24 | 98 | 1.53 | 7.0e-05 |
| MA0499.1 |  | hlh-11 (0.51) lin-32 hlh-14 hlh-1 | 99 | 1.52 | 7.3e-05 |
| Nkx2-5\_3436 |  | dsc-1 ceh-24 | 81 | 1.66 | 7.4e-05 |
| HOXA10\_2 |  | lin-39 php-3 pal-1 ceh-24 | 115 | 1.43 | 7.7e-05 |
| pTH10788 |  | tbx-33 | 108 | 1.46 | 7.9e-05 |
| HLF\_si |  | ces-2 atf-2 C48E7.11 F23F12.9 Y51H4A.4 | 94 | 1.55 | 8.2e-05 |
| CG31670\_SANGER\_5\_FBgn0031375 |  | CELE\_Y38H8A.5 | 161 | 1.22 | 8.3e-05 |
| pTH3477 |  | daf-16 | 128 | 1.36 | 8.6e-05 |
| V$TATA\_01 |  | tbp-1 | 84 | 1.62 | 8.9e-05 |
| pTH6641 |  | lin-31 | 92 | 1.56 | 9.3e-05 |
| pTH10722 |  | ref-2 (0.55) eor-1 egrh-3 | 60 | 1.88 | 9.8e-05 |
| MA0456.1 |  | ref-2 (0.55) T22C8.4 | 81 | 1.64 | 9.8e-05 |
| exd\_FlyReg\_FBgn0000611 |  | ceh-20 let-381 cfi-1 | 137 | 1.32 | 1.0e-04 |
| Sox17\_2837 |  | sox-4 | 128 | 1.36 | 1.0e-04 |
| pTH9279 |  | Y116A8C.22 | 132 | 1.34 | 1.1e-04 |
| pTH10650 |  | nhr-153 (0.72) | 107 | 1.46 | 1.2e-04 |
| ems\_FlyReg\_FBgn0000576 |  | ceh-2 (0.76) skn-1 | 97 | 1.52 | 1.2e-04 |
| pTH9297 |  | ceh-18 | 115 | 1.42 | 1.2e-04 |
| Pou3f4\_3773 |  | ceh-6 | 91 | 1.56 | 1.2e-04 |
| Tcf1\_2666 |  | hmbx-1 | 63 | 1.83 | 1.2e-04 |
| pTH8399 |  | lin-54 | 90 | 1.56 | 1.2e-04 |
| pTH5914 |  | attf-1 | 72 | 1.72 | 1.3e-04 |
| V$FREAC7\_01 |  | lin-31 | 128 | 1.35 | 1.4e-04 |
| pTH5922 |  | ceh-24 | 71 | 1.73 | 1.4e-04 |
| FOXK1\_1 |  | nhr-213 (0.71) lin-31 | 104 | 1.47 | 1.5e-04 |
| V$E47\_02 |  | lin-32 hlh-2 | 83 | 1.61 | 1.6e-04 |
| MA0470.1 |  | efl-1 F49E12.6 | 100 | 1.49 | 1.6e-04 |
| pTH10031 |  | mbr-1 | 68 | 1.75 | 1.6e-04 |
| pTH8216 |  | Y116A8C.22 | 77 | 1.66 | 1.6e-04 |
| Pou3f3\_3235 |  | ceh-6 | 45 | 2.14 | 1.6e-04 |
| BARHL2\_3 |  | ceh-1 lin-39 ceh-31 ceh-43 ceh-14 | 125 | 1.36 | 1.6e-04 |
| pTH9709 |  | die-1 | 134 | 1.32 | 1.8e-04 |
| NR2E3\_f1 |  | lin-39 lin-1 nhr-100 | 126 | 1.35 | 1.8e-04 |
| MA0264.1 |  | ceh-22 (0.75) dsc-1 ceh-24 | 64 | 1.79 | 1.8e-04 |
| V$RORA2\_01 |  | nhr-213 (0.71) nhr-118 | 66 | 1.77 | 1.8e-04 |
| pTH2283 |  | odd-2 (0.77) | 73 | 1.69 | 1.9e-04 |
| GM12878\_PBX3\_HudsonAlpha |  | ceh-20 dro-1 lin-31 nfya-2 | 83 | 1.60 | 2.0e-04 |
| pTH8996 |  | daf-8 sma-4 hlh-8 | 116 | 1.40 | 2.0e-04 |
| V$AREB6\_02 |  | ztf-6 | 63 | 1.79 | 2.1e-04 |
| pTH3510 |  | F13H6.1 (0.65) nhr-177 (0.58) nhr-2 nhr-86 | 77 | 1.65 | 2.1e-04 |
| MA0483.1 |  | lim-6 odd-1 | 100 | 1.48 | 2.3e-04 |
| Pou3f1\_3819 |  | ceh-6 | 87 | 1.56 | 2.4e-04 |
| pTH9177 |  | hsf-1 F10B5.3 Y53C10A.3 | 122 | 1.36 | 2.5e-04 |
| MA0118.1 |  | ref-2 (0.55) | 78 | 1.63 | 2.7e-04 |
| MA0386.1 |  | che-1 K11D2.4 tbp-1 | 108 | 1.43 | 2.8e-04 |
| V$HOX13\_01 |  | lin-39 | 74 | 1.66 | 2.9e-04 |
| V$AREB6\_01 |  | ztf-6 tbx-39 | 87 | 1.55 | 2.9e-04 |
| gl\_FlyReg\_FBgn0004618 |  | ceh-24 ces-1 T22H9.4 C34H4.5 | 113 | 1.40 | 3.0e-04 |
| pTH5423 |  | klf-2 (0.74) | 91 | 1.52 | 3.2e-04 |
| MA0163.1 |  | Y53H1A.2 (-0.66) D1081.8 | 24 | 3.04 | 3.2e-04 |
| pTH5169 |  | cfi-1 | 110 | 1.41 | 3.2e-04 |
| Mw160 |  | nhr-68 | 117 | 1.38 | 3.4e-04 |
| pTH9137 |  | nhr-65 (0.68) | 122 | 1.35 | 3.4e-04 |
| V$ZIC1\_01 |  | ref-2 (0.55) ZC328.2 (0.52) ztf-14 lin-1 | 79 | 1.61 | 3.4e-04 |
| pTH10816 |  | dmd-6 | 119 | 1.37 | 3.4e-04 |
| pTH10768 |  | med-2 | 142 | 1.27 | 3.5e-04 |
| pTH6591 |  | lin-31 | 119 | 1.37 | 3.7e-04 |
| EN1\_2 |  | ceh-16 | 45 | 2.06 | 3.7e-04 |
| pTH9380 |  | mel-28 | 147 | 1.25 | 3.8e-04 |
| I$SN\_02 |  | unc-120 K02D7.2 C24A1.2 | 109 | 1.41 | 3.8e-04 |
| MA0222.1 |  | ceh-20 ceh-32 F55C5.11 | 154 | 1.22 | 4.2e-04 |
| Sox1\_2631 |  | sox-4 | 104 | 1.43 | 4.2e-04 |
| Sox1\_4 |  | F56D1.1 gei-3 sox-4 | 84 | 1.56 | 4.3e-04 |
| TFE3\_f1 |  | pax-1 (0.74) hlh-30 (0.67) mdl-1 aha-1 | 57 | 1.83 | 4.3e-04 |
| pTH9928 |  | fos-1 (0.81) jun-1 sknr-1 crh-1 F45H11.6 | 106 | 1.42 | 4.4e-04 |
| pTH10714 |  | nhr-216 (0.52) nhr-84 nhr-142 | 93 | 1.49 | 4.7e-04 |
| pTH10638 |  | dmd-3 C34D1.1 | 85 | 1.54 | 4.9e-04 |
| V$PAX5\_02 |  | pax-3 pax-2 | 98 | 1.46 | 4.9e-04 |
| CG4854\_SANGER\_10\_FBgn0038766 |  | K11D2.4 | 116 | 1.37 | 5.0e-04 |
| Tcf1\_2666 |  | hmbx-1 | 61 | 1.77 | 5.0e-04 |
| pTH10645 |  | nhr-7 (0.87) elt-1 nhr-100 | 105 | 1.42 | 5.0e-04 |
| Lhx4\_1719 |  | lim-6 ceh-16 lim-7 | 100 | 1.45 | 5.2e-04 |
| V$GATA1\_03 |  | elt-1 alr-1 | 89 | 1.51 | 5.3e-04 |
| pTH6071 |  | C46E10.8 C33G8.2 | 93 | 1.49 | 5.5e-04 |
| pTH9256 |  | ceh-18 | 112 | 1.39 | 5.8e-04 |
| pnr\_SANGER\_5\_FBgn0003117 |  | elt-1 | 128 | 1.31 | 6.1e-04 |
| ZNF75A\_1 |  | lag-1 ztf-3 F26F4.8 | 105 | 1.42 | 6.3e-04 |
| pTH9245 |  | ceh-18 | 109 | 1.39 | 6.8e-04 |
| pTH5005 |  | crh-1 W08E12.1 attf-1 | 88 | 1.51 | 6.8e-04 |
| srp\_SANGER\_5\_FBgn0003507 |  | ceh-34 (0.62) elt-6 (0.54) elt-1 elt-3 ceh-32 elt-7 egl-27 | 96 | 1.46 | 7.2e-04 |
| pTH9924 |  | nhr-46 (0.64) | 107 | 1.40 | 7.3e-04 |
| pTH3998 |  | tbx-39 | 98 | 1.45 | 7.8e-04 |
| pTH9393 |  | ZC416.1 | 68 | 1.66 | 7.8e-04 |
| pTH10696 |  | Y44A6D.3 dpy-27 | 50 | 1.90 | 7.9e-04 |
| pTH5714 |  | nhr-239 | 96 | 1.46 | 8.1e-04 |
| HLH27 |  | lin-22 hlh-27 hlh-28 | 73 | 1.62 | 8.1e-04 |
| MA0007.2 |  | nhr-255 lin-14 | 102 | 1.42 | 8.4e-04 |
| IRX2\_1 |  | irx-1 | 105 | 1.41 | 8.5e-04 |
| V$CEBPA\_01 |  | C48E7.11 | 94 | 1.46 | 8.7e-04 |
| pTH9911 |  | fos-1 (0.81) atf-5 crh-1 | 78 | 1.57 | 9.0e-04 |
| amos\_da\_SANGER\_10\_FBgn0003270 |  | hlh-32 hlh-8 hlh-15 hlh-12 ngn-1 | 96 | 1.45 | 9.0e-04 |
| pTH10823 |  | B0310.2 | 93 | 1.47 | 9.8e-04 |
| MA0482.1 |  | elt-1 ztf-29 | 119 | 1.34 | 1.0e-03 |
| pTH9381 |  | ceh-18 | 106 | 1.40 | 1.0e-03 |
| ETS2\_f1 |  | lin-1 C24A1.2 | 100 | 1.43 | 1.0e-03 |
| pTH9043 |  | sem-2 (0.67) | 65 | 1.68 | 1.0e-03 |
| Ets97D\_SANGER\_10\_FBgn0004510 |  | lin-1 C24A1.2 | 119 | 1.34 | 1.1e-03 |
| pTH3046 |  | Y116A8C.22 | 85 | 1.51 | 1.1e-03 |
| pTH3064 |  | crh-1 | 32 | 2.34 | 1.1e-03 |
| pTH5250 |  | C48E7.11 | 107 | 1.39 | 1.1e-03 |
| HeLa-S3\_ZNF274\_UCD |  | C28G1.4 | 112 | 1.37 | 1.1e-03 |
| Hoxa6\_1040 |  | lin-39 | 96 | 1.45 | 1.1e-03 |
| Prrx2\_3072 |  | ceh-45 (0.55) ceh-1 lin-39 npax-3 ceh-12 ceh-43 alr-1 | 102 | 1.41 | 1.1e-03 |
| ZBTB49\_1 |  | C46E10.9 aha-1 | 97 | 1.43 | 1.2e-03 |
| Hoxb9\_3413 |  | pal-1 ceh-24 | 145 | 1.24 | 1.3e-03 |
| pTH10647 |  | nhr-232 | 96 | 1.44 | 1.3e-03 |
| Fer1\_da\_SANGER\_10\_FBgn0037475 |  | lin-39 lin-32 ceh-32 | 95 | 1.44 | 1.3e-03 |
| Tcf7\_0950 |  | pop-1 | 118 | 1.34 | 1.3e-03 |
| V$BRN2\_01 |  | ceh-18 | 134 | 1.27 | 1.3e-03 |
| MSX1\_3 |  | ceh-1 lin-39 ceh-9 ceh-31 ceh-43 alr-1 ceh-16 lim-7 | 96 | 1.44 | 1.3e-03 |
| FEV\_f1 |  | lin-1 C24A1.2 | 109 | 1.37 | 1.4e-03 |
| pTH2936 |  | nhr-239 | 81 | 1.52 | 1.5e-03 |
| ATF1\_si |  | crh-1 | 90 | 1.47 | 1.5e-03 |
| pTH6486 |  | nhr-145 | 101 | 1.41 | 1.5e-03 |
| V$LYF1\_01 |  | mbr-1 F26F4.8 | 100 | 1.41 | 1.5e-03 |
| FOXB1\_1 |  | lin-31 nhr-68 F55C5.11 | 87 | 1.48 | 1.6e-03 |
| TCF4\_2 |  | hlh-2 ztf-6 | 91 | 1.45 | 1.7e-03 |
| pTH3751 |  | tbx-39 | 87 | 1.48 | 1.8e-03 |
| pTH10013 |  | nhr-168 (0.61) | 108 | 1.37 | 1.8e-03 |
| pTH4325 |  | ceh-18 | 118 | 1.33 | 1.8e-03 |
| CEBPE\_f1 |  | C48E7.11 R07H5.10 | 104 | 1.39 | 1.8e-03 |
| Tbp\_pr781 |  | tbp-1 | 118 | 1.33 | 1.9e-03 |
| pTH2820 |  | ZC328.2 (0.52) | 86 | 1.48 | 1.9e-03 |
| pTH5118 |  | cfi-1 | 122 | 1.31 | 2.0e-03 |
| Pou2f3\_3986 |  | ceh-18 | 104 | 1.38 | 2.1e-03 |
| MA0452.2 |  | B0310.2 | 113 | 1.34 | 2.2e-03 |
| Hoxd11\_3873 |  | ceh-13 php-3 | 69 | 1.60 | 2.2e-03 |
| pTH9220 |  | mbr-1 | 77 | 1.53 | 2.3e-03 |
| pTH9163 |  | nhr-3 (0.6) | 106 | 1.37 | 2.3e-03 |
| RFX4\_2 |  | daf-19 | 78 | 1.52 | 2.4e-03 |
| Pou2f1\_3081 |  | ceh-18 | 82 | 1.50 | 2.4e-03 |
| pTH10805 |  | ztf-16 (0.84) | 77 | 1.53 | 2.5e-03 |
| Gsh2\_3990 |  | ceh-31 | 86 | 1.47 | 2.5e-03 |
| Irx6\_2623 |  | irx-1 | 92 | 1.43 | 2.5e-03 |
| pTH9173 |  | efl-2 | 74 | 1.55 | 2.6e-03 |
| MA0083.2 |  | unc-120 hlh-10 | 95 | 1.42 | 2.6e-03 |
| MA0161.1 |  | nfi-1 F49E12.6 | 94 | 1.42 | 2.6e-03 |
| Hoxa7\_2668 |  | lin-39 | 88 | 1.46 | 2.6e-03 |
| I$E74A\_01 |  | nhr-19 lin-1 C24A1.2 | 116 | 1.32 | 2.8e-03 |
| pTH5119 |  | cfi-1 | 102 | 1.38 | 2.8e-03 |
| pTH8983 |  | tag-347 | 92 | 1.43 | 2.9e-03 |
| pTH5812 |  | ceh-14 | 81 | 1.49 | 2.9e-03 |
| V$FOXJ2\_02 |  | lin-31 | 102 | 1.38 | 3.0e-03 |
| Smad3\_3805 |  | daf-8 | 153 | 1.19 | 3.1e-03 |
| GATA4\_1 |  | elt-1 end-3 | 98 | 1.40 | 3.2e-03 |
| Cart1\_1275 |  | alr-1 lim-7 | 95 | 1.41 | 3.2e-03 |
| pTH5018 |  | fos-1 (0.81) atf-7 crh-1 atf-6 C27D6.4 | 94 | 1.41 | 3.2e-03 |
| pTH10798 |  | Y75B8A.6 | 80 | 1.49 | 3.3e-03 |
| V$HEN1\_01 |  | hlh-15 | 81 | 1.49 | 3.3e-03 |
| Hlxb9\_3422 |  | ceh-12 | 85 | 1.46 | 3.4e-03 |
| Hoxb5\_3122 |  | lin-39 | 85 | 1.46 | 3.5e-03 |
| pTH9244 |  | tbx-39 | 93 | 1.42 | 3.5e-03 |
| Irx5\_2385 |  | irx-1 | 86 | 1.45 | 3.5e-03 |
| Mf28 |  | elt-1 | 102 | 1.37 | 3.5e-03 |
| MA0454.1 |  | odd-2 (0.77) odd-1 | 93 | 1.41 | 3.6e-03 |
| Dlx1\_1741 |  | ceh-43 | 93 | 1.41 | 3.7e-03 |
| pTH1001 |  | dnj-17 | 88 | 1.44 | 3.8e-03 |
| pTH10034 |  | nhr-66 (0.78) | 89 | 1.43 | 3.9e-03 |
| K562\_ZBTB7A\_HudsonAlpha |  | ZC328.2 (0.52) | 49 | 1.78 | 3.9e-03 |
| V$OCT1\_03 |  | ceh-18 | 92 | 1.42 | 4.0e-03 |
| Dlx2\_2273 |  | ceh-43 | 92 | 1.41 | 4.1e-03 |
| V$FAC1\_01 |  | gei-8 | 131 | 1.26 | 4.2e-03 |
| pTH8991 |  | cey-3 | 83 | 1.46 | 4.2e-03 |
| pTH9387 |  | C34D1.1 | 42 | 1.89 | 4.2e-03 |
| Nkx6-1\_2825 |  | cog-1 | 77 | 1.50 | 4.4e-03 |
| pTH5916 |  | efl-2 | 75 | 1.51 | 4.4e-03 |
| pTH6478 |  | lim-7 | 89 | 1.43 | 4.5e-03 |
| Hoxb8\_3780 |  | lin-39 | 89 | 1.42 | 4.8e-03 |
| Hoxb7\_3953 |  | lin-39 ceh-18 ceh-6 | 91 | 1.41 | 4.9e-03 |
| Tcf7l2\_3461 |  | pop-1 | 141 | 1.22 | 4.9e-03 |
| pTH9182 |  | tbx-39 | 90 | 1.42 | 5.0e-03 |
| V$GATA6\_01 |  | elt-1 | 86 | 1.44 | 5.1e-03 |
| Prop1\_3949 |  | ceh-53 (0.58) ceh-16 | 85 | 1.44 | 5.2e-03 |
| pTH5539 |  | unc-120 | 94 | 1.40 | 5.2e-03 |
| pTH9934 |  | Y53H1A.2 (-0.66) | 73 | 1.52 | 5.2e-03 |
| PAX8\_f1 |  | pax-2 | 70 | 1.54 | 5.2e-03 |
| Hmbox1\_2674 |  | hmbx-1 | 74 | 1.51 | 5.3e-03 |
| pTH8598 |  | odr-7 lin-1 nhr-273 nhr-79 | 154 | 1.18 | 5.3e-03 |
| pTH1014 |  | atf-5 | 141 | 1.22 | 5.3e-03 |
| V$PAX5\_01 |  | pax-2 F45H11.6 | 84 | 1.45 | 5.4e-03 |
| YY1\_1 |  | lsy-2 | 93 | 1.40 | 5.4e-03 |
| Hoxa5\_3415 |  | lin-39 | 86 | 1.43 | 5.7e-03 |
| Barx2\_3447 |  | ceh-43 | 89 | 1.42 | 5.9e-03 |
| pTH6423 |  | pha-2 (0.64) | 45 | 1.80 | 6.1e-03 |
| pTH10623 |  | scrt-1 | 98 | 1.37 | 6.2e-03 |
| Mw144 |  | elt-1 | 101 | 1.36 | 6.2e-03 |
| pTH6482 |  | ceh-19 (0.71) | 83 | 1.44 | 6.3e-03 |
| pTH10654 |  | ceh-90 lsy-27 | 49 | 1.74 | 6.4e-03 |
| Meox1\_2310 |  | ceh-31 | 90 | 1.41 | 6.5e-03 |
| pTH5778 |  | egl-5 | 80 | 1.46 | 6.8e-03 |
| Bsx\_3483 |  | ceh-31 | 93 | 1.39 | 6.9e-03 |
| pTH9135 |  | pop-1 | 122 | 1.27 | 6.9e-03 |
| pTH10030 |  | xbp-1 | 79 | 1.46 | 7.0e-03 |
| MA0262.1 |  | mab-3 | 58 | 1.62 | 7.0e-03 |
| pTH8985 |  | athp-1 | 88 | 1.41 | 7.0e-03 |
| CUX1\_2 |  | ceh-48 (-0.54) | 135 | 1.23 | 7.0e-03 |
| pTH5924 |  | nhr-255 | 107 | 1.33 | 7.1e-03 |
| V$RFX1\_02 |  | daf-19 | 90 | 1.40 | 7.2e-03 |
| HXA7\_f1 |  | lin-39 | 80 | 1.46 | 7.2e-03 |
| V$CDPCR3\_01 |  | ceh-48 (-0.54) ceh-24 | 159 | 1.16 | 7.2e-03 |
| Gata5\_3768 |  | elt-1 | 65 | 1.56 | 7.3e-03 |
| MCR\_f1 |  | nhr-255 | 100 | 1.35 | 7.3e-03 |
| pTH6327 |  | dsc-1 | 88 | 1.41 | 7.5e-03 |
| pTH3819 |  | ceh-18 | 78 | 1.46 | 7.8e-03 |
| Hoxb4\_2627 |  | lin-39 | 91 | 1.39 | 8.2e-03 |
| V$GR\_Q6 |  | nhr-255 | 86 | 1.41 | 8.9e-03 |
| V$IK2\_01 |  | F26F4.8 | 99 | 1.35 | 8.9e-03 |
| CDC5L\_si |  | irx-1 D1081.8 | 83 | 1.43 | 9.2e-03 |
| MA0544.1 |  | lin-22 gei-11 | 70 | 1.51 | 9.2e-03 |
| TBX3\_f1 |  | ceh-45 (0.55) tbx-39 | 105 | 1.33 | 9.3e-03 |
| Irx3\_0920 |  | irx-1 | 42 | 1.81 | 9.3e-03 |
| pTH5082 |  | atf-2 | 152 | 1.17 | 9.6e-03 |
| Hoxb3\_1720 |  | lin-39 | 86 | 1.40 | 1.0e-02 |
| pTH10777 |  | dmd-3 | 99 | 1.35 | 1.0e-02 |
| MA0126.1 |  | lin-48 (0.64) pax-3 | 58 | 1.60 | 1.0e-02 |
| Elf3 |  | C24A1.2 | 112 | 1.30 | 1.1e-02 |
| tgo\_ss\_SANGER\_5\_FBgn0015014 |  | aha-1 | 81 | 1.43 | 1.1e-02 |
| pTH8998 |  | mab-3 | 101 | 1.33 | 1.1e-02 |
| Jundm2\_0911 |  | fos-1 (0.81) skn-1 | 97 | 1.35 | 1.2e-02 |
| Gata3\_1024 |  | elt-1 | 90 | 1.38 | 1.2e-02 |
| HES1\_f1 |  | lin-22 | 84 | 1.41 | 1.2e-02 |
| MA0095.2 |  | lsy-2 | 130 | 1.23 | 1.2e-02 |
| pTH6516 |  | F19F10.1 C24A1.2 | 99 | 1.34 | 1.2e-02 |
| pTH10640 |  | dmd-4 ceh-32 | 81 | 1.42 | 1.2e-02 |
| Tcf3\_3787 |  | pop-1 | 138 | 1.21 | 1.2e-02 |
| pTH6268 |  | ceh-2 (0.76) | 55 | 1.61 | 1.2e-02 |
| Nkx6-3\_3446 |  | cog-1 | 74 | 1.46 | 1.2e-02 |
| Elf3\_3876 |  | C24A1.2 | 114 | 1.28 | 1.3e-02 |
| Mafk\_3106 |  | F45H11.6 | 116 | 1.28 | 1.3e-02 |
| Spdef |  | lin-1 | 87 | 1.39 | 1.3e-02 |
| pTH9215 |  | C34D1.1 | 65 | 1.52 | 1.3e-02 |
| pTH10718 |  | egl-43 (0.67) | 94 | 1.36 | 1.3e-02 |
| pTH9282 |  | attf-1 | 65 | 1.52 | 1.3e-02 |
| V$GATA1\_02 |  | elt-1 | 78 | 1.43 | 1.3e-02 |
| pTH6106 |  | nhr-182 (0.79) | 80 | 1.42 | 1.4e-02 |
| pTH9300 |  | dmd-3 C34D1.1 | 66 | 1.51 | 1.4e-02 |
| Barhl1\_1 |  | ceh-31 | 83 | 1.40 | 1.5e-02 |
| pTH10772 |  | ceh-52 | 153 | 1.16 | 1.5e-02 |
| pTH9969 |  | pag-3 | 109 | 1.29 | 1.5e-02 |
| CXXC1\_si |  | F52B11.1 F21D5.4 | 81 | 1.41 | 1.5e-02 |
| pTH9198 |  | dmd-3 | 61 | 1.54 | 1.5e-02 |
| HIF1A\_si |  | hif-1 | 71 | 1.47 | 1.5e-02 |
| Elf5 |  | C24A1.2 | 96 | 1.34 | 1.5e-02 |
| pTH9165 |  | ztf-27 (0.9) | 150 | 1.17 | 1.5e-02 |
| V$TCF11\_01 |  | skn-1 | 84 | 1.40 | 1.5e-02 |
| pTH5257 |  | C48E7.11 | 106 | 1.30 | 1.6e-02 |
| pTH9884 |  | tbx-39 | 45 | 1.71 | 1.6e-02 |
| Hoxa7\_3750 |  | lin-39 | 89 | 1.37 | 1.6e-02 |
| pTH6562 |  | ceh-5 | 83 | 1.40 | 1.6e-02 |
| pTH10028 |  | nhr-204 (0.82) | 64 | 1.51 | 1.6e-02 |
| pTH6449 |  | ceh-43 | 82 | 1.40 | 1.7e-02 |
| Vsx1\_1728 |  | alr-1 | 88 | 1.37 | 1.7e-02 |
| pTH9958 |  | ztf-6 | 123 | 1.24 | 1.7e-02 |
| V$YY1\_01 |  | lsy-2 | 122 | 1.25 | 1.7e-02 |
| pTH8649 |  | mbr-1 | 109 | 1.29 | 1.7e-02 |
| V$S8\_01 |  | ceh-45 (0.55) | 99 | 1.32 | 1.7e-02 |
| Hoxc4\_3491 |  | lin-39 | 89 | 1.36 | 1.8e-02 |
| Hmx1\_3423 |  | ceh-9 | 165 | 1.13 | 1.8e-02 |
| V$OCT1\_06 |  | ztf-9 ceh-18 | 106 | 1.29 | 2.0e-02 |
| MA0260.1 |  | che-1 ces-1 | 93 | 1.34 | 2.0e-02 |
| OTX2\_si |  | ceh-53 (0.58) ceh-45 (0.55) alr-1 ceh-36 | 93 | 1.34 | 2.0e-02 |
| Dlx3\_1030 |  | ceh-43 | 88 | 1.36 | 2.0e-02 |
| pTH1049 |  | elt-1 | 62 | 1.51 | 2.0e-02 |
| pTH2684 |  | fos-1 (0.81) | 93 | 1.34 | 2.1e-02 |
| pTH9384 |  | cfi-1 | 110 | 1.28 | 2.1e-02 |
| Hoxa9\_2622 |  | lin-39 php-3 | 95 | 1.33 | 2.2e-02 |
| Lhx1\_2240 |  | lim-7 | 92 | 1.34 | 2.2e-02 |
| pTH9076 |  | C01G12.1 | 49 | 1.63 | 2.3e-02 |
| Six4\_2860 |  | ceh-32 | 45 | 1.67 | 2.3e-02 |
| V$PAX2\_02 |  | pax-1 (0.74) | 80 | 1.39 | 2.4e-02 |
| pTH3712 |  | D1081.8 | 139 | 1.19 | 2.4e-02 |
| V$GATA3\_01 |  | elt-1 | 37 | 1.79 | 2.4e-02 |
| PAX5\_si |  | pax-2 | 89 | 1.35 | 2.5e-02 |
| pTH9925 |  | ztf-11 | 109 | 1.28 | 2.5e-02 |
| pTH6447 |  | ceh-19 (0.71) | 47 | 1.63 | 2.6e-02 |
| pTH3997 |  | C04F5.9 | 78 | 1.39 | 2.8e-02 |
| Nsy-7 |  | nsy-7 | 77 | 1.40 | 2.9e-02 |
| ECC-1\_ERALPHA\_HudsonAlpha |  | nhr-71 (0.51) nhr-6 | 47 | 1.63 | 2.9e-02 |
| Hoxd13\_2356 |  | pal-1 | 68 | 1.45 | 2.9e-02 |
| Cdx2\_4272 |  | ceh-13 | 125 | 1.22 | 3.0e-02 |
| Nkx1-1\_3856 |  | ceh-30 | 85 | 1.36 | 3.1e-02 |
| pTH5928 |  | ceh-34 (0.62) | 45 | 1.64 | 3.1e-02 |
| Elf4 |  | C24A1.2 | 90 | 1.34 | 3.1e-02 |
| pTH8863 |  | hmg-12 (-0.76) | 132 | 1.20 | 3.1e-02 |
| Gmeb1\_1745 |  | attf-1 | 65 | 1.46 | 3.2e-02 |
| Lbx2\_3869 |  | mls-2 | 143 | 1.17 | 3.5e-02 |
| Nkx1-2\_3214 |  | ceh-30 | 76 | 1.39 | 3.5e-02 |
| pTH9907 |  | nhr-34 (0.8) | 118 | 1.24 | 3.5e-02 |
| Zfp161\_2858 |  | pzf-1 | 36 | 1.76 | 3.5e-02 |
| Hoxd1\_3448 |  | ceh-12 | 95 | 1.31 | 3.5e-02 |
| pTH6569 |  | ceh-43 | 84 | 1.35 | 3.6e-02 |
| KLF8\_f1 |  | klf-1 | 75 | 1.39 | 3.6e-02 |
| pTH6445 |  | ceh-5 | 100 | 1.29 | 3.7e-02 |
| pTH8318 |  | attf-1 | 52 | 1.55 | 3.7e-02 |
| MA0027.1 |  | ceh-16 | 151 | 1.15 | 3.9e-02 |
| MA0139.1 |  | F58G1.2 | 79 | 1.37 | 3.9e-02 |
| pTH5078 |  | ces-2 | 80 | 1.36 | 4.0e-02 |
| Emx2\_3420 |  | ceh-2 (0.76) | 48 | 1.58 | 4.1e-02 |
| TLX1\_f1 |  | ceh-19 (0.71) | 77 | 1.38 | 4.2e-02 |
| pTH9974 |  | hlh-16 | 86 | 1.33 | 4.3e-02 |
| Vax1\_3499 |  | C02F12.10 | 82 | 1.35 | 4.4e-02 |
| Tcfap2a\_2337 |  | aptf-1 | 46 | 1.59 | 4.5e-02 |
| pTH9080 |  | mnm-2 | 84 | 1.34 | 4.7e-02 |
| Pbx1\_3203 |  | ceh-20 | 98 | 1.29 | 4.8e-02 |
| Hoxa11\_2218 |  | php-3 | 91 | 1.31 | 4.8e-02 |
| Hoxa4\_3426 |  | lin-39 | 85 | 1.33 | 4.8e-02 |
| Hnf4a\_2640 |  | nhr-62 (0.83) | 136 | 1.18 | 4.9e-02 |
| pTH9915 |  | zip-3 | 56 | 1.49 | 5.0e-02 |

### Correlated (and anti-correlated) transcription factors

|  |  |
| --- | --- |
| **Transcription factor** | **Correlation** |
| peb-1 | 0.93 |
| nhr-56 | 0.92 |
| nhr-123 | 0.91 |
| nhr-103 | 0.91 |
| nhr-98 | 0.91 |
| ztf-27 | 0.90 |
| nhr-18 | 0.90 |
| nhr-133 | 0.89 |
| nhr-7 | 0.87 |
| nhr-203 | 0.87 |
| nhr-201 | 0.87 |
| nhr-202 | 0.87 |
| nhr-112 | 0.87 |
| nhr-237 | 0.86 |
| nhr-70 | 0.86 |
| nhr-117 | 0.86 |
| nhr-42 | 0.86 |
| nhr-129 | 0.86 |
| nhr-128 | 0.85 |
| nhr-136 | 0.85 |
| nhr-61 | 0.84 |
| ztf-16 | 0.84 |
| nhr-62 | 0.83 |
| fkh-9 | 0.83 |
| nhr-130 | 0.83 |
| R144.3 | -0.55 |
| cey-2 | -0.56 |
| W02D7.6 | -0.56 |
| vab-3 | -0.56 |
| lim-4 | -0.57 |
| mxl-1 | -0.57 |
| D2030.7 | -0.58 |
| sptf-1 | -0.58 |
| zag-1 | -0.59 |
| dhhc-7 | -0.61 |
| nfyc-1 | -0.62 |
| bed-1 | -0.63 |
| unc-3 | -0.64 |
| T07F8.4 | -0.64 |
| lir-3 | -0.64 |
| zip-4 | -0.66 |
| Y53H1A.2 | -0.66 |
| F52B5.7 | -0.66 |
| repo-1 | -0.68 |
| ztf-4 | -0.68 |
| ceh-41 | -0.69 |
| C09F5.3 | -0.73 |
| hmg-12 | -0.76 |
| F21D5.9 | -0.76 |
| him-8 | -0.83 |

### ChIP peaks enriched

|  |  |  |  |  |
| --- | --- | --- | --- | --- |
| **Gene** | **Experiment** | **Number of upstream peaks** | **Enrichment** | **FDR corrected p** |
| pha-4 | PHA-4\_Larvae-L2-stage | 97 | 2.04 | 1.1e-12 |
| nhr-23 | NHR-23\_Larvae-L3-stage | 42 | 2.52 | 1.3e-06 |
| pha-4 | PHA-4\_Larvae-L4-stage | 55 | 2.10 | 2.8e-06 |
| nhr-25 | NHR-25\_Larvae-L2-stage | 63 | 1.83 | 2.4e-05 |
| W03F9.2 | W03F9.2\_L4-Young-Adult-stage-larvae | 75 | 1.65 | 6.4e-05 |
| nhr-28 | NHR-28\_Larvae-L4-stage | 44 | 2.08 | 9.6e-05 |
| C01B12.2 | C01B12.2\_Larvae-L2-stage | 86 | 1.54 | 1.1e-04 |
| ham-1 | HAM-1\_Larvae-L4-stage | 72 | 1.62 | 2.3e-04 |
| dpl-1 | DPL-1\_Larvae-L4-stage | 41 | 2.08 | 2.3e-04 |
| hpl-2 | HPL-2\_Fed-L1-stage-larvae | 39 | 2.06 | 4.7e-04 |
| fos-1 | FOS-1\_Larvae-L2-stage | 80 | 1.51 | 7.1e-04 |
| lsy-2 | LSY-2\_Larvae-L1-stage | 77 | 1.51 | 1.3e-03 |
| nhr-77 | NHR-77\_Larvae-L4-stage | 88 | 1.43 | 1.5e-03 |
| peb-1 | PEB-1\_Larvae-L2-stage | 20 | 2.80 | 1.6e-03 |
| dve-1 | DVE-1\_Late-Embryos | 28 | 2.28 | 1.7e-03 |
| ceh-26 | CEH-26\_Late-Embryonic-stage | 48 | 1.74 | 2.9e-03 |
| ceh-38 | CEH-38\_Larvae-L3-stage | 26 | 2.24 | 4.0e-03 |
| R02D3.7 | R02D3.7\_Larvae-L3-stage | 34 | 1.92 | 6.4e-03 |
| sax-3 | SAX-3\_Larvae-L4-stage | 70 | 1.45 | 1.1e-02 |
| ham-1 | HAM-1\_Fed-L1-stage-larvae | 63 | 1.49 | 1.2e-02 |
| sem-4 | SEM-4\_Larvae-L2-stage | 63 | 1.49 | 1.2e-02 |
| jun-1 | JUN-1\_Larvae-L3-stage | 20 | 2.34 | 1.4e-02 |
| dpl-1 | DPL-1\_Fed-L1-stage-larvae | 23 | 2.12 | 2.0e-02 |
| C16A3.4 | C16A3.4\_Fed-L1-stage-larvae | 3 | 13.61 | 4.4e-02 |
| fos-1 | FOS-1\_Fed-L1-stage-larvae | 26 | 1.86 | 4.9e-02 |
| fos-1 | FOS-1\_Larvae-L4-stage | 29 | 1.79 | 4.9e-02 |
